# Preoperative Thyroid Hormone Ratios and Complete Blood Count (CBC)-Derived Biomarkers for Differentiating Benign Thyroid Nodules from Papillary Thyroid Carcinoma: A Retrospective Single-Center Case–Control Study

**DOI:** 10.3390/jcm15155913

**Published:** 2026-07-29

**Authors:** Neşe Bülbül, Deniz Gülnihal, Rukiye Çiftçi, Ebru Sena Poyraz, Özgür Eken, Ayşe Fulya Özkanlı, Katja Weiss, Thomas Rosemann, Beat Knechtle

**Affiliations:** 1 Department of Endocrinology and Metabolism, Gaziantep Islam Science and Technology University, Gaziantep 27000, Türkiye; drnesebulbul@yahoo.com.tr; 2 Department of Physiotherapy and Rehabilitation, Faculty of Health Sciences, Erzurum Technical University, Erzurum 25100, Türkiye; gulnihal.deniz@erzurum.edu.tr; 3 Department of Anatomy, Gaziantep Islam Science and Technology University, Gaziantep 27000, Türkiye; rukiyekelesciftci@hotmail.com (R.Ç.); ebrusena.calisir@gibtu.edu.tr (E.S.P.); 4 Department of Physical Education and Sport Teaching, Faculty of Sports Sciences, Inonu University, Malatya 44280, Türkiye; ozgur.eken@inonu.edu.tr; 5 Department of Public Health, Gaziantep Islam Science and Technology University, Gaziantep 27000, Türkiye; dr.fulyaalben@gmail.com; 6 Institute of Primary Care, University of Zurich, 8091 Zurich, Switzerland; katja@weiss.co.com (K.W.); thomas.rosemann@usz.ch (T.R.); 7 Medbase St. Gallen Am Vadianplatz, Vadianstrasse 26, 9001 St. Gallen, Switzerland

**Keywords:** thyroid nodules, papillary thyroid carcinoma, thyroid-stimulating hormone, platelet-to-lymphocyte ratio, thyroid hormone ratios, complete blood count-derived markers

## Abstract

**Background/Objectives:** Preoperative differentiation between benign thyroid nodules and papillary thyroid carcinoma (PTC) remains clinically challenging, particularly after indeterminate cytology. This study evaluated routinely available complete blood count (CBC)-derived indices, erythrocyte parameters, thyroid function tests, and arithmetic thyroid hormone ratios. **Methods:** This retrospective, single-center case–control study reviewed thyroidectomy records from July 2020 to November 2024. Histopathology was re-adjudicated from the source reports; 158 adults with clearly benign nodules (*n* = 93) or PTC (*n* = 65) formed the final analytic cohort. Missing laboratory values were handled by variable-specific complete-case analysis. Benjamini–Hochberg false-discovery-rate (FDR) correction and an exploratory multivariable logistic model were applied. **Results:** Preoperative TSH and the conventional platelet-to-lymphocyte ratio (PLR) were higher in PTC (*p* = 0.002, q = 0.013; and *p* = 0.004, q = 0.023), whereas the fT4/TSH and fT3/TSH ratios were lower (both q = 0.013). The fT3 difference was nominal (*p* = 0.018, q = 0.076), and MCV was not significant (*p* = 0.076). In 126 complete cases, each doubling of TSH was associated with higher odds of PTC (adjusted odds ratio [aOR] 1.51, 95% CI 1.08–2.13), whereas higher fT3 was associated with lower odds (aOR 0.41, 95% CI 0.19–0.87). **Conclusions:** TSH and fT3 were independently associated with PTC in the exploratory model, while the PLR and TSH-normalized hormone ratios differed between groups but lacked validated diagnostic performance. These measures should not be used in isolation; prospective multicenter studies are needed to determine whether they add clinically meaningful information to ultrasonographic, cytological, and molecular assessments.

## 1. Introduction

Thyroid cancer is the most prevalent malignancy of the endocrine system and represents a significant global public health concern owing to its rapidly increasing incidence [1]. According to current epidemiological data, thyroid cancer is the seventh most common malignancy worldwide [1]. Long-term analyses have revealed that this increase in thyroid cancer incidence is particularly associated with a marked rise in cases of papillary thyroid carcinoma (PTC) [2]. PTC is the predominant histological subtype of thyroid malignancies, accounting for approximately 85–90% of all thyroid cancers [1,3]. In most cases, papillary thyroid carcinoma is diagnosed following the detection of a thyroid nodule during routine clinical evaluation or as an incidental finding [4].

Thyroid nodules are common in clinical practice, with a prevalence of approximately 3–7% on palpation and 20–70% in the general population when detected by high-resolution ultrasonography [5,6]. Although the vast majority of thyroid nodules are benign, only a small proportion harbor malignant lesions, such as papillary thyroid carcinoma [3]. However, the presence of overlapping ultrasonographic and cytological features between certain benign nodules and papillary thyroid carcinoma complicates the preoperative differentiation between benign and malignant lesions [3,7,8]. Although ultrasonography and fine-needle aspiration biopsy (FNA) are the cornerstone diagnostic modalities for evaluating thyroid nodules, their diagnostic accuracy remains limited in a subset of cases [3]. Notably, approximately 20–30% of FNA specimens are classified as Bethesda category III or IV, which cannot be definitively categorized as benign or malignant and are therefore referred to as ‘indeterminate cytology’ in the literature [3,8,9].

Although molecular testing contributes to the assessment of malignancy risk, its widespread application in large populations is limited by high costs, limited availability, and a lack of suitability for routine clinical use [3,9,10,11]. Consequently, there is a growing need for low-cost, easily applicable, and objective biomarkers to aid in the preoperative differentiation of benign and malignant thyroid lesions. It has long been recognized that inflammation plays a pivotal role in cancer pathogenesis, and tumor-supporting inflammatory processes are now regarded as one of the fundamental biological hallmarks of cancer [12]. This recognition has heightened interest in cost-effective, readily accessible, and easily measurable inflammatory parameters in routine clinical practice [13]. In this context, numerous studies have demonstrated that CBC-based indices [including the neutrophil-to-lymphocyte ratio (NLR), platelet-to-lymphocyte ratio (PLR), lymphocyte-to-monocyte ratio (LMR), red blood cell distribution width (RDW), and hemoglobin-to-RDW (Hb/RDW) ratio] are associated with tumor progression, aggressive biological behavior, and survival outcomes across a variety of solid malignancies [13,14,15,16,17]. In particular, the Hb/RDW ratio has been reported to possess prognostic value as a biomarker reflecting cancer-related inflammation, anemia, tumor burden, and alterations in the tumor microenvironment. This ratio has been shown to correlate with tumor stage and aggressive phenotypes in several malignancies, most notably colorectal cancer [13,14]. In particular, the hemoglobin-to-red blood cell distribution width (Hb/RDW) ratio is markedly influenced by cancer-related physiological alterations. Tumor-induced systemic stress and inflammatory responses can modify this ratio by affecting hematological parameters, such as hemoglobin and RDW. Chronic inflammation associated with malignancy disrupts erythropoiesis and erythrocyte maturation, resulting in an increased RDW, whereas tumor-related anemia leads to reduced hemoglobin levels. The combined impact of these opposing processes can produce substantial changes in the Hb/RDW ratio [13]. Furthermore, previous studies have demonstrated a negative correlation between the Hb/RDW ratio and tumor stage in patients with colorectal cancer, with lower Hb/RDW values being associated with advanced disease and more aggressive tumor behavior [14].

In the context of thyroid disease, several studies have reported that the RDW is significantly elevated in malignant thyroid nodules compared with benign lesions and may serve as a valuable indicator of nodule biological behavior [18,19]. Additionally, evidence suggests that the NLR is significantly elevated in cases of papillary thyroid microcarcinoma and PTC. The PLR is associated with lateral cervical lymph node metastasis in PTC, particularly among female patients. Furthermore, the LMR has been identified as a significant predictor of recurrence in PTC [15,16,17].

Studies systematically evaluating the Hb/RDW ratio for differentiating benign thyroid nodules from PTC remain limited. The present study therefore aimed to compare preoperative CBC-derived inflammatory indices (RDW, Hb/RDW, NLR, PLR, and LMR), erythrocyte parameters including MCV, thyroid function tests, and arithmetic thyroid hormone ratios (fT3/fT4, fT4/TSH, and fT3/TSH) between patients with histopathologically confirmed benign thyroid nodules and PTC. The analyses were intended to identify exploratory group-level associations that may inform future diagnostic model development, rather than to establish standalone diagnostic cutoffs.

## 2. Materials and Methods

### 2.1. Study Design and Setting

This retrospective, observational, single-center case–control study used routinely collected clinical, laboratory, surgical, and pathology records; no additional diagnostic or therapeutic procedures were performed for research purposes. This study was conducted at the Endocrinology and Metabolism Diseases Outpatient Clinic of Gaziantep Islam Science and Technology University Faculty of Medicine, Ersin Arslan Training and Research Hospital, Türkiye. Records from July 2020 through November 2024 were reviewed.

### 2.2. Study Population

**Figure 1:**
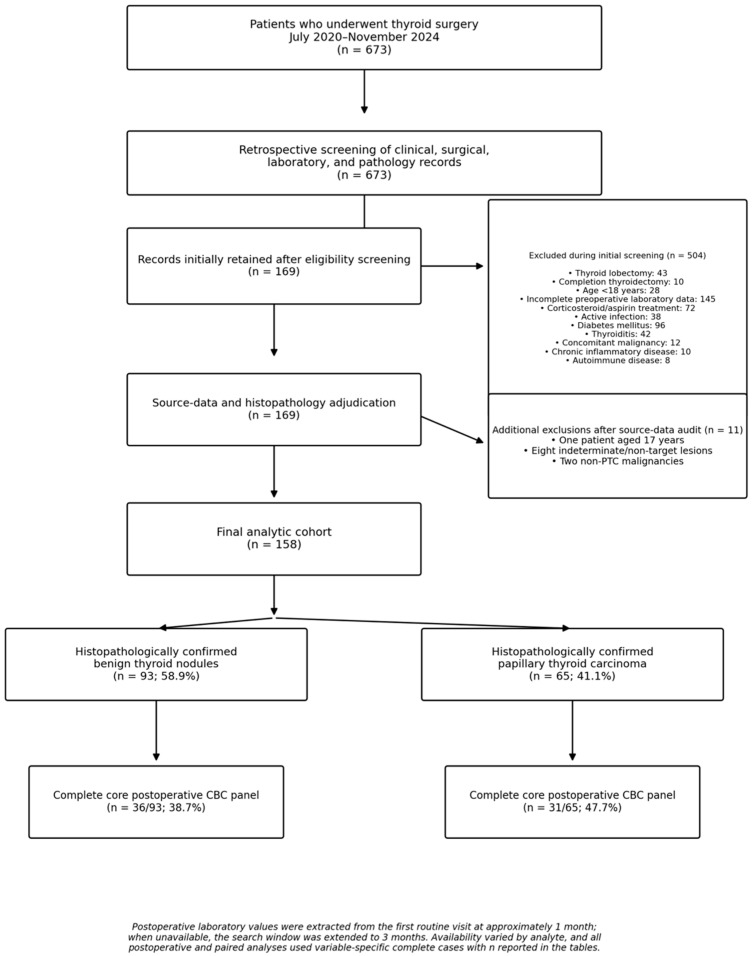
Flow diagram of initial eligibility screening, source-data and histopathology adjudication, final study-group allocation, and availability of complete core postoperative CBC panels.

The source population comprised patients evaluated at the Endocrinology Outpatient Clinic who underwent thyroid surgery for nodular or multinodular goiter. Initial eligibility criteria were age 18 years or older, total thyroidectomy, and availability of relevant preoperative laboratory and final pathology records. Exclusion criteria were thyroid lobectomy or completion thyroidectomy, age younger than 18 years, incomplete preoperative laboratory data, corticosteroid or aspirin treatment, active infection, diabetes mellitus, thyroiditis, concomitant malignancy, chronic inflammatory disease, or autoimmune disease. The original screening log recorded 673 surgical patients and 504 primary exclusions, leaving 169 records for source-data adjudication; the exclusion categories shown in Figure 1 sum to 504. During source-data verification, free-text histopathology reports were re-adjudicated. One additional record from a 17-year-old patient that had remained in the 169-record extract despite the age criterion, eight records with indeterminate or non-target lesions, and two records with non-PTC malignancies were excluded. The final analytic cohort therefore comprised 158 patients: 93 with histopathologically confirmed benign thyroid nodules and 65 with PTC. Five inconsistent group-code entries were corrected according to the final pathology report. No separate healthy control group was included, and no age- or sex-matching procedure was performed. Because this study was a retrospective census of eligible records from the predefined period, no a priori sample-size calculation was performed. Preoperative data availability varied by analyte; no values were imputed, and variable-specific complete-case sample sizes are reported in all tables. Postoperative laboratory values were extracted from the first routine follow-up visit at approximately 1 month after surgery; when a 1-month value was unavailable, the search window was extended to 3 months. Availability therefore varied by analyte.

### 2.3. Laboratory Measurements

Demographic variables, including age and sex, were recorded for all participants. Preoperative laboratory values were obtained from routine analyses performed before surgery. Postoperative values represented the first available routine result at approximately 1 month, with extension of the retrieval window to 3 months when the 1-month result was absent. Following thyroidectomy, the surgeon typically initiated levothyroxine at 75–100 micrograms/day, most commonly 100 micrograms/day, and recommended review at approximately 1 month. Because treatment adherence and dose titration varied, achievement of euthyroidism could take longer in some patients; postoperative analyses were therefore considered secondary and exploratory rather than standardized treatment-effect analyses.

The recorded CBC and related measures included hemoglobin, hematocrit, platelet count, RDW expressed as standard deviation (RDW-SD) and coefficient of variation (RDW-CV), MCV, differential leukocyte measures, C-reactive protein, and serum albumin. Complete blood count parameters were measured using an XN-1000 automated hematology analyzer (Sysmex Corporation, Kobe, Japan). Serum C-reactive protein and albumin concentrations were measured using an AU5800 automated clinical chemistry analyzer (Beckman Coulter, Inc., Brea, CA, USA). Albumin was standardized to g/L. Two source entries recorded as 3.9 and 4.5 g/dL were converted to 39 and 45 g/L, respectively, to harmonize the units with the remaining records. Parathyroid hormone was removed from comparative analyses because it was available for only a small, non-systematic subset and was not part of the prespecified study objective. The analyzer model and assay-specific reference interval information were not retained in the de-identified retrospective extract; all tests were performed in the hospital central laboratory under routine internal quality-control procedures.

CBC-derived indices were recalculated directly from the source variables. Hb/RDW-SD and Hb/RDW-CV are the hemoglobin value divided by RDW-SD and RDW-CV, respectively. The NLR is the neutrophil percentage divided by the lymphocyte percentage. The conventional PLR is the platelet count divided by the absolute lymphocyte count, and the LMR is the absolute lymphocyte count divided by the absolute monocyte count. The previous PLR calculation based on the lymphocyte percentage was corrected, and all affected tables and statistical analyses were recomputed.

Thyroid function was assessed using serum thyroid-stimulating hormone (TSH), free triiodothyronine (fT3), and free thyroxine (fT4) concentrations were measured using electrochemiluminescence immunoassays on a cobas e 601 analyzer (Roche Diagnostics GmbH, Mannheim, Germany). The fT3/fT4 ratio was calculated as fT3 (pg/mL) divided by fT4 (ng/dL); fT4/TSH and fT3/TSH were calculated from the raw concentrations without unit conversion. These are dimensioned arithmetic ratios and are not biologically standardized indices. Ratio values were calculated only when the denominator was strictly greater than zero. Source values recorded as 0.00 were retained for descriptive analysis of the original analyte but were excluded from ratio calculations when used as denominators.

### 2.4. Ethical Considerations

All data were obtained from routine clinical follow-up records, and no additional interventions or costs were imposed on the patients. The study protocol was approved by the institutional ethics committee (approval date: 26 May 2025; approval number: 2025/653.48.27). This study was conducted in accordance with the principles of the Declaration of Helsinki.

### 2.5. Statistical Analysis

Categorical variables are summarized as frequencies and percentages. Normality was assessed with the Shapiro–Wilk test. Between-group comparisons used Welch’s *t* test when both group distributions were approximately normal and the Mann–Whitney U test otherwise. Age was compared with Welch’s *t* test and sex with Yates’ continuity-corrected chi-square test. Paired preoperative–postoperative comparisons used the paired *t* test when the distribution of within-patient differences was approximately normal and the Wilcoxon signed-rank test otherwise. No missing values were imputed; each analysis used all available complete observations for the variables involved.

Effect sizes are reported as Cohen’s d for independent comparisons, d_z for paired *t* tests, and r for rank-based tests. Values of 0.2, 0.5, and 0.8 for d/d_z and 0.1, 0.3, and 0.5 for r were interpreted as small, moderate, and large, respectively. Two-sided *p* values were supplemented by Benjamini–Hochberg FDR-adjusted q values, calculated separately for the preoperative between-group, postoperative between-group, overall paired, and within-group paired analysis families. An exploratory multivariable logistic regression of complete cases included age, sex, MCV, log2-transformed TSH, fT3, and log2-transformed PLR; PTC was coded as 1 and benign nodules as 0. Observations with non-positive TSH or PLR values were not eligible for log2 transformation and were excluded from the complete-case model. The odds ratios for TSH and PLR therefore represent a doubling of the predictor. Arithmetic hormone ratios were not entered together with their component hormones because of collinearity. No diagnostic cutoff, sensitivity, specificity, calibration, or external validation analysis was performed. Statistical analyses were conducted using Python version 3.13 (Python Software Foundation, Wilmington, DE, USA), SciPy version 1.17.0 (SciPy Community, USA), and statsmodels version 0.14.6 (statsmodels Development Team, USA).

## 3. Results

A total of 158 patients were included after source-data and histopathology adjudication: 93 (58.9%) had benign thyroid nodules, and 65 (41.1%) had PTC. The mean age was 44.2 ± 11.7 years, and 134 patients (84.8%) were female. Age and sex did not differ significantly between groups (Table 1 and Table 2). Preoperative and postoperative availability varied by analyte; all tables report the exact n, and no imputation was used. Complete core postoperative CBC panels were available for 67 patients (36 benign and 31 PTC), whereas thyroid function and other postoperative measures had variable-specific sample sizes.

Descriptive preoperative, postoperative, and change values for hematological and inflammatory parameters and for thyroid-function measures and arithmetic thyroid-hormone ratios are summarized in Table 3A and Table 3B, respectively.

In preoperative comparisons, the median TSH level was higher in PTC than in benign nodules (1.76 vs. 1.19 µIU/mL; *p* = 0.002; q = 0.013; r = 0.27), and the conventional PLR was higher (157.73 vs. 125.49; *p* = 0.004; q = 0.023; r = 0.23). The median fT4/TSH and fT3/TSH ratios were lower in PTC (*p* = 0.001 for both; q = 0.013 for both; r = 0.28). The fT3 difference was nominal (*p* = 0.018; q = 0.076), while MCV was not significant (*p* = 0.076; q = 0.266) (Table 4). In an exploratory complete-case multivariable model (*n* = 126), each doubling of TSH was associated with higher odds of PTC (aOR 1.51, 95% CI 1.08–2.13; *p* = 0.017), and each 1 pg/mL increase in fT3 was associated with lower odds (aOR 0.41, 95% CI 0.19–0.87; *p* = 0.021). TSH and fT3 were the only covariates reaching nominal statistical significance; MCV and PLR were not independently significant.

Postoperative laboratory availability varied by analyte because follow-up testing was not uniform. Complete core postoperative CBC panels were available for 67 patients (36 benign and 31 PTC); individual postoperative measures ranged from *n* = 35 to *n* = 158. The only nominal between-group postoperative difference was platelet count (*p* = 0.023), but it did not remain significant after FDR correction (q = 0.482). No postoperative analyte showed an FDR-significant difference between the benign and PTC groups (Table 5).

Overall paired analyses used variable-specific complete pairs. After FDR correction, hemoglobin, fT3, Hb/RDW-SD, Hb/RDW-CV, fT3/fT4, fT4/TSH, and fT3/TSH decreased, whereas TSH increased (Table 6). These postoperative differences were observed within the 1–3-month retrieval window during levothyroxine initiation and titration and should not be interpreted as standardized physiological treatment effects.

Within-group analyses were also performed using variable-specific complete pairs (Table 7). FDR-significant postoperative changes were concentrated in thyroid function measures and hemoglobin. Benign-nodule patients additionally showed an FDR-significant platelet increase, whereas the PLR decreased in the PTC group. These findings remain secondary because follow-up timing, replacement dose, adherence, and dose titration were not standardized.

## 4. Discussion

This source-verified reanalysis compared routine preoperative hematological and thyroid-related measures between surgically treated patients with clearly benign thyroid nodules and histopathologically confirmed PTC. Correcting pathology-group coding, excluding non-target diagnoses, harmonizing albumin units, recalculating the conventional PLR, and applying FDR control materially changed the interpretation. A higher TSH and PLR and lower fT4/TSH and fT3/TSH remained significant after multiplicity correction, whereas the previously reported MCV difference did not.

The higher TSH observed in PTC is consistent with prior studies and meta-analytic evidence linking greater thyrotropin concentrations with malignancy risk among patients with thyroid nodules [20,21,22,23]. The effect was small, and substantial overlap remained between groups. In the exploratory adjusted model, the odds of PTC increased by approximately 51% for each doubling of TSH, independent of age, sex, MCV, fT3, and PLR. This association is not equivalent to diagnostic accuracy and should not be used to define a clinical cutoff.

Free T3 was nominally lower in PTC and remained inversely associated with PTC in the multivariable model, although its unadjusted comparison did not survive FDR correction. The fT4/TSH and fT3/TSH ratios remained lower after FDR correction. Because these dimensioned arithmetic ratios are strongly influenced by the denominator and are not physiologically standardized indices, they should be viewed as exploratory transformations rather than established biomarkers.

Recalculation of the PLR using absolute lymphocyte count produced higher conventional PLR values in PTC and an FDR-significant unadjusted difference. However, the PLR did not remain independently significant after adjustment. Prior reports have linked inflammatory indices to papillary microcarcinoma, nodal metastasis, recurrence, or prediction models in selected populations [15,16,17,24,25,26,27]. The present finding supports further evaluation of the PLR as a component of multivariable models, not as a standalone discriminator.

MCV was not significantly different after source-data correction and pathology adjudication (*p* = 0.076; q = 0.266). Iron status, vitamin B12, folate, and other nutritional variables were unavailable, preventing the assessment of common hematological confounders. Because assay-specific reference intervals were not retained in the de-identified extract, individual MCV values could not be formally classified as within or outside the laboratory reference range. The revised data therefore do not support an independent MCV association in this cohort.

RDW, Hb/RDW, NLR, and LMR did not show robust preoperative differences. This contrasts with thyroid-specific reports comparing PTC with healthy controls or evaluating selected clinicopathological outcomes [18,19,28]. The RDW has also been studied as a prognostic marker in other malignancies, including hepatocellular carcinoma [29], but such evidence does not establish thyroid-specific discrimination. A benign surgical-nodule control group is clinically more demanding than a healthy control group and may attenuate inflammatory contrasts. Negative findings in this setting help define the limited standalone utility of these indices.

Postoperative analyses covered the first available routine measurement at approximately 1 month, extending to 3 months when necessary. Levothyroxine was typically initiated at 75–100 micrograms/day, most commonly 100 micrograms/day. Variation in adherence and subsequent dose titration can delay euthyroidism. Consequently, the observed postoperative increases in TSH and decreases in fT3 and arithmetic ratios should be interpreted as non-standardized follow-up observations rather than causal effects of surgery or a uniform replacement regimen.

Clinically, none of the evaluated laboratory measures can replace ultrasonography, fine-needle aspiration cytology, or indicated molecular testing. Molecular and tissue markers, including emerging PTC-associated candidates such as SPP1, address different biological domains and require independent validation [30]. Future models may integrate routine laboratory variables with ultrasound risk categories, Bethesda cytology, and molecular tests for indeterminate nodules [10,11].

No ROC curve, cutoff, sensitivity, specificity, calibration, decision-curve, or external validation analysis was performed. Therefore, the results establish associations only and do not demonstrate incremental diagnostic benefit. Prospective multicenter studies should prespecify candidate variables, standardize assays and follow-up, retain complete nutritional and treatment data, and perform internal and external validation.

This study has several limitations. Its retrospective design introduces selection and information bias, and the cohort was restricted to patients undergoing total thyroidectomy. Laboratory availability varied by analyte and was handled by complete-case analysis, which may introduce additional selection bias. The sample was predominantly female and derived from a single center, limiting generalizability across sex, ethnicity, iodine status, and healthcare settings. Analyzer model and assay-specific reference intervals were unavailable in the de-identified extract; consequently, the MCV values could not be formally classified relative to laboratory-specific normal ranges. The multivariable model was exploratory and based on 126 complete cases. Although FDR correction was applied, residual confounding and model optimism remain possible. Postoperative timing, levothyroxine dose, adherence, and titration were not standardized.

The principal strength of the revision is the direct source-data audit: final pathology was used as the reference standard, non-target diagnoses were excluded, group-code inconsistencies were corrected, albumin units were harmonized, the standard PLR was recomputed, zero denominators were handled explicitly, and every analysis reports its actual sample size.

Taken together, the findings support cautious investigation of TSH, PLR, and arithmetic TSH-denominator ratios as candidate features in future validated models while arguing against their current use as isolated diagnostic tests.

## 5. Conclusions

In this retrospective, source-verified surgical cohort, higher preoperative TSH and conventional PLR values and lower fT4/TSH and fT3/TSH ratios remained associated with PTC after FDR correction. Higher TSH and lower fT3 values retained independent associations in an exploratory multivariable model, whereas the MCV and PLR did not. The substantial overlap between groups, variable-specific missingness, retrospective design, and absence of validated diagnostic performance analyses preclude clinical cutoff or standalone diagnostic use. Prospective multicenter validation within integrated ultrasound, cytology, and molecular models is required.

## Figures and Tables

**Table 1:** Demographic and clinical characteristics of the patients.

Variable	*n*	%
Age (years), mean ± SD	44.20 ± 11.73	
Age (years), min–max	22–75	
Sex, *n* (%)		
Male	24	15.2
Female	134	84.8
Pathological diagnosis, *n* (%)		
Benign thyroid nodule	93	58.9
Papillary thyroid carcinoma	65	41.1

SD, standard deviation; *n*, number; %, percentage; min, minimum; max, maximum.

**Table 2:** Comparison of demographic characteristics between benign and malignant groups.

Variable	Benign (*n* = 93)	PTC (*n* = 65)	*p* Value
Age (years), mean ± SD	45.46 ± 11.61	42.38 ± 11.75	0.106
Sex, *n* (%)			0.285
Male	17 (18.3)	7 (10.8)	
Female	76 (81.7)	58 (89.2)	

SD, standard deviation; *n*, number; %, percentage. Continuous variables are presented as mean ± standard deviation, and categorical variables are presented as number and percentage. Age: Welch’s *t* test; sex: Yates’ continuity-corrected chi-square test.

**Table 3:** (**A**). Descriptive statistics of preoperative and postoperative hematological and inflammatory parameters. (**B**). Descriptive statistics of preoperative and postoperative thyroid function tests and arithmetic thyroid hormone ratios.

**(A)**						
**Parameter**	**Preoperative**		**Postoperative**		**Delta (Post-Pre)**	
	**Mean ± SD**	**Median (Min–Max)**	**Mean ± SD**	**Median (Min–Max)**	**Mean ± SD**	**Median (Min–Max)**
Hemoglobin (g/dL)	13.42 ± 1.80; *n* = 151	13.40 (8.00–18.40); *n* = 151	12.42 ± 1.61; *n* = 158	12.10 (8.30–18.90); *n* = 158	−0.94 ± 1.80; *n* = 151	−0.60 (−5.90–4.30); *n* = 151
Hematocrit (%)	39.55 ± 4.62; *n* = 151	39.70 (26.10–51.20); *n* = 151	39.13 ± 5.46; *n* = 67	39.30 (26.00–53.20); *n* = 67	−0.68 ± 3.55; *n* = 67	−0.40 (−11.10–8.80); *n* = 67
Platelet count (10^3^/µL)	283.9 ± 70.5; *n* = 151	283.0 (103.0–528.0); *n* = 151	286.2 ± 80.3; *n* = 67	268.0 (153.0–463.0); *n* = 67	4.5 ± 49.1; *n* = 67	3.0 (−114.0–147.0); *n* = 67
RDW-SD (fL)	41.76 ± 3.83; *n* = 151	41.60 (34.40–66.90); *n* = 151	42.55 ± 5.53; *n* = 67	41.60 (35.00–69.10); *n* = 67	0.65 ± 5.96; *n* = 67	−0.10 (−22.30–28.40); *n* = 67
RDW-CV (%)	14.61 ± 3.03; *n* = 151	13.90 (11.60–41.60); *n* = 151	14.70 ± 3.31; *n* = 67	13.90 (12.20–31.60); *n* = 67	0.43 ± 3.36; *n* = 67	0.10 (−10.30–17.80); *n* = 67
Neutrophils (%)	62.91 ± 10.47; *n* = 151	62.00 (25.20–92.40); *n* = 151	63.92 ± 12.03; *n* = 67	63.50 (42.80–89.40); *n* = 67	0.95 ± 11.17; *n* = 67	0.00 (−26.50–30.50); *n* = 67
Lymphocytes (%)	27.52 ± 8.75; *n* = 151	28.00 (5.50–45.90); *n* = 151	26.01 ± 10.37; *n* = 67	27.60 (5.10–49.10); *n* = 67	−1.78 ± 9.83; *n* = 67	−0.40 (−31.00–20.30); *n* = 67
C-reactive protein (mg/L)	5.40 ± 12.91; *n* = 158	2.75 (0.31–116.70); *n* = 158	13.63 ± 20.51; *n* = 41	4.80 (0.31–105.30); *n* = 41	4.25 ± 14.91; *n* = 41	0.27 (−57.00–47.54); *n* = 41
Albumin (g/L)	43.64 ± 3.83; *n* = 158	43.70 (28.90–53.40); *n* = 158	43.77 ± 4.11; *n* = 35	44.00 (32.30–50.20); *n* = 35	−0.50 ± 4.21; *n* = 35	−0.30 (−10.00–7.81); *n* = 35
Mean corpuscular volume (fL)	81.23 ± 6.49; *n* = 158	82.30 (61.60–95.90); *n* = 158	83.08 ± 5.58; *n* = 63	82.80 (67.50–94.60); *n* = 63	0.88 ± 3.63; *n* = 63	0.40 (−4.80–18.60); *n* = 63
Hb/RDW-SD ratio	0.32 ± 0.06; *n* = 151	0.33 (0.16–0.49); *n* = 151	0.32 ± 0.07; *n* = 67	0.33 (0.16–0.48); *n* = 67	−0.01 ± 0.04; *n* = 67	−0.01 (−0.15–0.09); *n* = 67
Hb/RDW-CV ratio	0.95 ± 0.21; *n* = 151	0.96 (0.40–1.59); *n* = 151	0.94 ± 0.23; *n* = 67	0.98 (0.41–1.55); *n* = 67	−0.04 ± 0.13; *n* = 67	−0.03 (−0.55–0.35); *n* = 67
Neutrophil-to-lymphocyte ratio	2.91 ± 2.41; *n* = 151	2.19 (0.95–16.80); *n* = 151	3.49 ± 3.18; *n* = 67	2.26 (0.87–17.53); *n* = 67	0.31 ± 3.23; *n* = 67	−0.03 (−9.38–13.40); *n* = 67
Platelet-to-lymphocyte ratio	167.69 ± 184.20; *n* = 151	135.29 (27.50–1930.00); *n* = 151	146.49 ± 62.89; *n* = 67	132.22 (62.41–481.43); *n* = 67	−46.72 ± 261.05; *n* = 67	−11.67 (−1724.55–299.43); *n* = 67
Lymphocyte-to-monocyte ratio	4.05 ± 1.80; *n* = 158	3.92 (0.40–14.00); *n* = 158	4.14 ± 2.08; *n* = 158	3.97 (0.47–16.17); *n* = 158	0.09 ± 2.13; *n* = 158	0.04 (−10.00–10.46); *n* = 158
**(B)**						
	**Preoperative**		**Postoperative**		**Delta (Post-Pre)**	
**Parameter**	**Mean ± SD**	**Median (Min-Max)**	**Mean ± SD**	**Median (Min-Max)**	**Mean ± SD**	**Median (Min-Max)**
Thyroid-stimulating hormone (µIU/mL)	1.69 ± 1.44; *n* = 133	1.36 (0.00–11.31); *n* = 133	10.59 ± 15.75; *n* = 84	3.48 (0.02–79.70); *n* = 84	9.18 ± 15.83; *n* = 72	2.08 (−2.49–78.09); *n* = 72
Free triiodothyronine (pg/mL)	3.59 ± 1.14; *n* = 131	3.41 (2.21–14.73); *n* = 131	2.80 ± 0.63; *n* = 79	2.83 (1.50–5.41); *n* = 79	−0.88 ± 1.49; *n* = 68	−0.72 (−11.44–1.04); *n* = 68
Free thyroxine (ng/dL)	1.60 ± 2.07; *n* = 130	1.04 (0.54–11.10); *n* = 130	1.98 ± 2.71; *n* = 86	1.13 (0.00–13.80); *n* = 86	0.06 ± 3.11; *n* = 73	0.02 (−9.99–13.13); *n* = 73
fT3/fT4 ratio	3.35 ± 1.35; *n* = 127	3.28 (0.37–8.22); *n* = 127	2.59 ± 1.40; *n* = 78	2.50 (0.23–10.52); *n* = 78	−0.70 ± 2.04; *n* = 66	−0.71 (−7.63–6.31); *n* = 66
fT4/TSH ratio	3.25 ± 11.77; *n* = 129	0.71 (0.08–107.00); *n* = 129	6.86 ± 26.72; *n* = 83	0.33 (0.00–164.29); *n* = 83	0.69 ± 27.63; *n* = 69	−0.51 (−106.77–152.86); *n* = 69
fT3/TSH ratio	10.37 ± 50.87; *n* = 128	2.47 (0.24–480.00); *n* = 128	6.21 ± 20.64; *n* = 76	0.68 (0.03–153.50); *n* = 76	−3.29 ± 46.43; *n* = 63	−2.21 (−323.65–141.89); *n* = 63

Values are shown with the exact variable-specific n. Delta summaries include only patients with both preoperative and postoperative measurements. Albumin is reported in g/L; PLR uses absolute lymphocyte count. SD, standard deviation; fT3, free triiodothyronine; fT4, free thyroxine; TSH, thyroid-stimulating hormone. Ratios were calculated only with strictly positive denominators.

**Table 4:** Preoperative laboratory comparisons between benign thyroid nodules and papillary thyroid carcinoma.

Parameter	Benign (*n* = 93)	PTC (*n* = 65)	*p* Value; FDR q	Effect Size
Hemoglobin (g/dL)	13.60 (8.00–18.40) (*n* = 90)	13.20 (8.60–16.20) (*n* = 61)	0.381; q = 0.800	r = 0.07
Hematocrit (%)	39.90 (26.10–51.20) (*n* = 90)	39.00 (27.70–46.60) (*n* = 61)	0.531; q = 0.826	r = 0.05
Platelet count (10^3^/µL)	286.0 (103.0–528.0) (*n* = 90)	275.0 (183.0–470.0) (*n* = 61)	0.684; q = 0.844	r = 0.03
RDW-SD (fL)	41.60 (34.40–51.20) (*n* = 90)	41.10 (34.60–66.90) (*n* = 61)	0.337; q = 0.800	r = 0.08
RDW-CV (%)	13.70 (11.60–41.60) (*n* = 90)	13.90 (12.20–26.70) (*n* = 61)	0.918; q = 0.964	r = 0.01
Neutrophils (%)	62.45 (25.20–92.40) (*n* = 90)	61.80 (43.20–90.20) (*n* = 61)	0.611; q = 0.826	r = 0.04
Lymphocytes (%)	27.23 ± 8.59 (*n* = 90)	27.94 ± 9.03 (*n* = 61)	0.630; q = 0.826	d = 0.08
C-reactive protein (mg/L)	3.00 (0.31–116.70) (*n* = 93)	2.54 (0.33–26.24) (*n* = 65)	0.377; q = 0.800	r = 0.07
Albumin (g/L)	43.60 (28.90–53.10) (*n* = 93)	44.00 (36.80–53.40) (*n* = 65)	0.975; q = 0.975	r = 0.00
Mean corpuscular volume (fL)	82.80 (61.60–95.90) (*n* = 93)	81.40 (66.80–93.80) (*n* = 65)	0.076; q = 0.266	r = 0.14
Thyroid-stimulating hormone (µIU/mL)	1.19 (0.00–11.31) (*n* = 76)	1.76 (0.24–5.56) (*n* = 57)	0.002; q = 0.013	r = 0.27
Free triiodothyronine (pg/mL)	3.44 (2.21–14.73) (*n* = 77)	3.30 (2.30–4.73) (*n* = 54)	0.018; q = 0.076	r = 0.21
Free thyroxine (ng/dL)	1.08 (0.64–11.10) (*n* = 74)	0.96 (0.54–9.35) (*n* = 56)	0.184; q = 0.552	r = 0.12
Hb/RDW-SD ratio	0.33 ± 0.06 (*n* = 90)	0.32 ± 0.05 (*n* = 61)	0.543; q = 0.826	d = 0.10
Hb/RDW-CV ratio	0.96 (0.41–1.59) (*n* = 90)	0.95 (0.40–1.28) (*n* = 61)	0.530; q = 0.826	r = 0.05
Neutrophil-to-lymphocyte ratio	2.20 (1.05–16.80) (*n* = 90)	2.11 (0.95–11.55) (*n* = 61)	0.581; q = 0.826	r = 0.04
Platelet-to-lymphocyte ratio	125.49 (27.50–1930.00) (*n* = 90)	157.73 (48.26–408.75) (*n* = 61)	0.004; q = 0.023	r = 0.23
Lymphocyte-to-monocyte ratio	3.80 (0.40–11.33) (*n* = 93)	4.00 (1.60–14.00) (*n* = 65)	0.849; q = 0.938	r = 0.02
fT3/fT4 ratio	3.34 (0.37–6.95) (*n* = 73)	3.24 (0.41–8.22) (*n* = 54)	0.749; q = 0.874	r = 0.03
fT4/TSH ratio	1.02 (0.08–107.00) (*n* = 73)	0.59 (0.15–8.35) (*n* = 56)	0.001; q = 0.013	r = 0.28
fT3/TSH ratio	3.00 (0.24–480.00) (*n* = 74)	1.95 (0.64–13.17) (*n* = 54)	0.001; q = 0.013	r = 0.28

Values are the mean ± SD for Welch’s *t* tests and median (min–max) for Mann–Whitney U tests. q values are Benjamini–Hochberg FDR-adjusted within the preoperative comparison family. Effect sizes: Cohen’s d for Welch’s *t* tests and r for Mann–Whitney U tests. The PLR is calculated as the platelet count divided by the absolute lymphocyte count.

**Table 5:** Postoperative laboratory comparisons between benign thyroid nodules and papillary thyroid carcinoma.

Parameter	Benign (*n* = 93)	PTC (*n* = 65)	*p* Value; FDR q	Effect Size
Hemoglobin (g/dL)	12.10 (8.80–18.90) (*n* = 93)	12.30 (8.30–17.50) (*n* = 65)	0.629; q = 1.000	r = 0.04
Hematocrit (%)	38.88 ± 5.44 (*n* = 36)	39.42 ± 5.57 (*n* = 31)	0.691; q = 1.000	d = 0.10
Platelet count (10^3^/µL)	306.3 ± 87.4 (*n* = 36)	262.8 ± 65.0 (*n* = 31)	0.023; q = 0.482	d = 0.56
RDW-SD (fL)	41.05 (35.00–64.30) (*n* = 36)	42.00 (37.20–69.10) (*n* = 31)	1.000; q = 1.000	r = 0.00
RDW-CV (%)	14.00 (12.20–26.60) (*n* = 36)	13.80 (12.40–31.60) (*n* = 31)	0.469; q = 1.000	r = 0.09
Neutrophils (%)	64.40 ± 11.97 (*n* = 36)	63.36 ± 12.28 (*n* = 31)	0.728; q = 1.000	d = 0.09
Lymphocytes (%)	25.76 ± 10.46 (*n* = 36)	26.31 ± 10.43 (*n* = 31)	0.829; q = 1.000	d = 0.05
C-reactive protein (mg/L)	3.45 (0.31–105.30) (*n* = 24)	6.10 (0.38–32.00) (*n* = 17)	0.771; q = 1.000	r = 0.05
Albumin (g/L)	43.70 (37.20–49.70) (*n* = 16)	44.10 (32.30–50.20) (*n* = 19)	0.842; q = 1.000	r = 0.03
Mean corpuscular volume (fL)	83.11 ± 5.14 (*n* = 34)	83.06 ± 6.15 (*n* = 29)	0.970; q = 1.000	d = 0.01
Thyroid-stimulating hormone (µIU/mL)	3.62 (0.06–79.70) (*n* = 56)	3.01 (0.02–64.91) (*n* = 28)	0.857; q = 1.000	r = 0.02
Free triiodothyronine (pg/mL)	2.67 (1.50–5.41) (*n* = 54)	2.94 (1.78–4.03) (*n* = 25)	0.379; q = 1.000	r = 0.10
Free thyroxine (ng/dL)	1.10 (0.00–10.61) (*n* = 58)	1.17 (0.21–13.80) (*n* = 28)	0.678; q = 1.000	r = 0.04
Hb/RDW-SD ratio	0.32 ± 0.07 (*n* = 36)	0.32 ± 0.06 (*n* = 31)	0.877; q = 1.000	d = 0.04
Hb/RDW-CV ratio	1.01 (0.43–1.55) (*n* = 36)	0.93 (0.41–1.31) (*n* = 31)	0.407; q = 1.000	r = 0.10
Neutrophil-to-lymphocyte ratio	2.43 (0.98–17.53) (*n* = 36)	2.16 (0.87–13.68) (*n* = 31)	0.576; q = 1.000	r = 0.07
Platelet-to-lymphocyte ratio	136.08 (62.41–481.43) (*n* = 36)	122.17 (74.56–234.29) (*n* = 31)	0.133; q = 1.000	r = 0.18
Lymphocyte-to-monocyte ratio	4.00 (0.47–16.17) (*n* = 93)	3.86 (1.09–11.09) (*n* = 65)	0.397; q = 1.000	r = 0.07
fT3/fT4 ratio	2.53 (0.23–4.67) (*n* = 53)	2.48 (0.27–10.52) (*n* = 25)	0.940; q = 1.000	r = 0.01
fT4/TSH ratio	0.32 (0.00–164.29) (*n* = 56)	0.33 (0.00–153.33) (*n* = 27)	0.911; q = 1.000	r = 0.01
fT3/TSH ratio	0.60 (0.03–52.22) (*n* = 52)	0.82 (0.05–153.50) (*n* = 24)	0.733; q = 1.000	r = 0.04

Values are mean ± SD or median (min–max), as determined by the distribution. Exact n is shown in each group cell. q values are FDR-adjusted within the postoperative comparison family. Effect sizes are Cohen’s d for Welch’s *t* tests and r for Mann–Whitney U tests.

**Table 6:** Variable-specific paired comparisons of preoperative and postoperative laboratory parameters.

Parameter	Preoperative	Postoperative	*p* Value; FDR q	Effect Size
Hemoglobin (g/dL) (*n* = 151)	13.40 (8.00–18.40)	12.10 (8.30–18.90)	<0.001; q = <0.001	r = 0.48
Hematocrit (%) (*n* = 67)	40.20 (27.40–50.70)	39.30 (26.00–53.20)	0.219; q = 0.384	r = 0.15
Platelet count (10^3^/µL) (*n* = 67)	281.6 ± 69.3	286.2 ± 80.3	0.454; q = 0.579	d_z = 0.09
RDW-SD (fL) (*n* = 67)	41.10 (36.80–66.90)	41.60 (35.00–69.10)	0.659; q = 0.729	r = 0.05
RDW-CV (%) (*n* = 67)	13.60 (11.60–26.70)	13.90 (12.20–31.60)	0.295; q = 0.476	r = 0.13
Neutrophils (%) (*n* = 67)	62.97 ± 11.11	63.92 ± 12.03	0.490; q = 0.579	d_z = 0.08
Lymphocytes (%) (*n* = 67)	29.50 (5.50–45.40)	27.60 (5.10–49.10)	0.352; q = 0.528	r = 0.11
C-reactive protein (mg/L) (*n* = 41)	3.30 (0.33–116.70)	4.80 (0.31–105.30)	0.045; q = 0.106	r = 0.31
Albumin (g/L) (*n* = 35)	44.27 ± 3.83	43.77 ± 4.11	0.486; q = 0.579	d_z = 0.12
Mean corpuscular volume (fL) (*n* = 63)	83.40 (64.20–93.80)	82.80 (67.50–94.60)	0.216; q = 0.384	r = 0.16
Thyroid-stimulating hormone (µIU/mL) (*n* = 72)	1.22 (0.00–4.39)	3.66 (0.02–79.70)	<0.001; q = <0.001	r = 0.69
Free triiodothyronine (pg/mL) (*n* = 68)	3.35 (2.54–14.73)	2.85 (1.50–4.03)	<0.001; q = <0.001	r = 0.75
Free thyroxine (ng/dL) (*n* = 73)	1.11 (0.54–11.10)	1.15 (0.00–13.80)	0.987; q = 0.987	r = 0.00
Hb/RDW-SD ratio (*n* = 67)	0.34 (0.16–0.49)	0.33 (0.16–0.48)	0.018; q = 0.046	r = 0.29
Hb/RDW-CV ratio (*n* = 67)	0.99 (0.40–1.59)	0.98 (0.41–1.55)	0.009; q = 0.031	r = 0.32
Neutrophil-to-lymphocyte ratio (*n* = 67)	2.09 (0.95–16.80)	2.26 (0.87–17.53)	0.496; q = 0.579	r = 0.08
Platelet-to-lymphocyte ratio (*n* = 67)	131.82 (27.50–1930.00)	132.22 (62.41–481.43)	0.120; q = 0.252	r = 0.19
Lymphocyte-to-monocyte ratio (*n* = 158)	3.92 (0.40–14.00)	3.97 (0.47–16.17)	0.741; q = 0.778	r = 0.03
fT3/fT4 ratio (*n* = 66)	3.18 (0.37–8.22)	2.46 (0.23–10.52)	<0.001; q = <0.001	r = 0.47
fT4/TSH ratio (*n* = 69)	1.01 (0.19–107.00)	0.23 (0.00–153.33)	0.010; q = 0.031	r = 0.31
fT3/TSH ratio (*n* = 63)	2.99 (0.69–326.00)	0.65 (0.03–153.50)	<0.001; q = 0.001	r = 0.46

Paired *t* tests were used for approximately normal differences and Wilcoxon signed-rank tests otherwise. Exact paired n is shown for each parameter. q values are FDR-adjusted within the overall paired family.

**Table 7:** Exploratory within-group paired comparisons in the benign thyroid nodule and papillary thyroid carcinoma groups.

Parameter	Benign			PTC		
	Preoperative	Postoperative	*p*; q; Effect	Preoperative	Postoperative	*p*; q; Effect
Hemoglobin (g/dL)	13.60 (8.00–18.40) (*n* = 90)	12.10 (8.80–18.90)	<0.001/<0.001/r = 0.54	13.25 ± 1.53 (*n* = 61)	12.60 ± 1.70	0.002/0.019/d_z = 0.42
Hematocrit (%)	39.06 ± 4.56 (*n* = 36)	38.88 ± 5.44	0.724/0.838/d_z = 0.06	41.00 (27.70–46.60) (*n* = 31)	39.30 (26.30–48.10)	0.206/0.393/r = 0.23
Platelet count (10^3^/µL)	283.5 ± 82.6 (*n* = 36)	306.3 ± 87.4	0.001/0.005/d_z = 0.58	279.5 ± 50.9 (*n* = 31)	262.8 ± 65.0	0.080/0.210/d_z = 0.33
RDW-SD (fL)	40.90 (36.80–51.20) (*n* = 36)	41.05 (35.00–64.30)	0.242/0.423/r = 0.20	41.10 (37.30–66.90) (*n* = 31)	42.00 (37.20–69.10)	0.621/0.725/r = 0.09
RDW-CV (%)	13.55 (11.60–17.60) (*n* = 36)	14.00 (12.20–26.60)	0.198/0.378/r = 0.22	13.70 (12.20–26.70) (*n* = 31)	13.80 (12.40–31.60)	0.758/0.758/r = 0.06
Neutrophils (%)	64.51 ± 10.41 (*n* = 36)	64.40 ± 11.97	0.948/0.948/d_z = 0.01	61.19 ± 11.79 (*n* = 31)	63.36 ± 12.28	0.355/0.567/d_z = 0.17
Lymphocytes (%)	26.39 ± 9.79 (*n* = 36)	25.76 ± 10.46	0.639/0.838/d_z = 0.08	29.42 ± 9.92 (*n* = 31)	26.31 ± 10.43	0.146/0.307/d_z = 0.27
C-reactive protein (mg/L)	3.18 (0.88–116.70) (*n* = 24)	3.45 (0.31–105.30)	0.376/0.607/r = 0.18	3.40 (0.33–11.90) (*n* = 17)	6.10 (0.38–32.00)	0.039/0.138/r = 0.50
Albumin (g/L)	44.66 ± 3.60 (*n* = 16)	44.12 ± 3.66	0.407/0.611/d_z = 0.21	43.95 ± 4.08 (*n* = 19)	43.48 ± 4.52	0.705/0.752/d_z = 0.09
Mean corpuscular volume (fL)	83.35 (64.20–89.70) (*n* = 34)	83.60 (69.70–91.40)	0.138/0.289/r = 0.26	83.50 (67.50–93.80) (*n* = 29)	82.00 (67.50–94.60)	0.482/0.619/r = 0.13
Thyroid-stimulating hormone (µIU/mL)	0.95 (0.00–3.14) (*n* = 45)	3.79 (0.06–79.70)	<0.001/<0.001/r = 0.74	1.34 (0.28–4.39) (*n* = 27)	3.52 (0.02–64.91)	0.004/0.028/r = 0.57
Free triiodothyronine (pg/mL)	3.41 (2.69–14.73) (*n* = 44)	2.71 (1.50–3.82)	<0.001/<0.001/r = 0.85	3.37 ± 0.55 (*n* = 24)	2.88 ± 0.60	0.005/0.028/d_z = 0.63
Free thyroxine (ng/dL)	1.14 (0.64–11.10) (*n* = 46)	1.10 (0.00–10.61)	0.694/0.838/r = 0.06	1.08 (0.54–9.35) (*n* = 27)	1.20 (0.21–13.80)	0.501/0.619/r = 0.13
Hb/RDW-SD ratio	0.33 ± 0.06 (*n* = 36)	0.32 ± 0.07	0.053/0.123/d_z = 0.33	0.33 ± 0.06 (*n* = 31)	0.32 ± 0.06	0.126/0.293/d_z = 0.28
Hb/RDW-CV ratio	0.98 ± 0.21 (*n* = 36)	0.95 ± 0.23	0.050/0.123/d_z = 0.34	1.01 (0.40–1.28) (*n* = 31)	0.93 (0.41–1.31)	0.060/0.180/r = 0.34
Neutrophil-to-lymphocyte ratio	2.37 (1.12–16.80) (*n* = 36)	2.43 (0.98–17.53)	0.925/0.948/r = 0.02	1.85 (0.95–11.55) (*n* = 31)	2.16 (0.87–13.68)	0.378/0.567/r = 0.16
Platelet-to-lymphocyte ratio	120.79 (27.50–1930.00) (*n* = 36)	136.08 (62.41–481.43)	0.451/0.631/r = 0.13	157.73 (76.47–356.92) (*n* = 31)	122.17 (74.56–234.29)	0.001/0.019/r = 0.58
Lymphocyte-to-monocyte ratio	3.80 (0.40–11.33) (*n* = 93)	4.00 (0.47–16.17)	0.758/0.838/r = 0.03	4.00 (1.60–14.00) (*n* = 65)	3.86 (1.09–11.09)	0.411/0.575/r = 0.10
fT3/fT4 ratio	3.04 (0.37–6.95) (*n* = 42)	2.46 (0.23–4.67)	0.004/0.011/r = 0.45	3.21 (2.08–8.22) (*n* = 24)	2.46 (0.27–10.52)	0.019/0.081/r = 0.49
fT4/TSH ratio	1.51 (0.23–107.00) (*n* = 43)	0.23 (0.00–26.79)	<0.001/0.003/r = 0.52	0.72 (0.19–8.35) (*n* = 26)	0.27 (0.00–153.33)	0.716/0.752/r = 0.07
fT3/TSH ratio	3.51 (0.95–326.00) (*n* = 40)	0.52 (0.03–40.43)	<0.001/0.001/r = 0.58	2.31 (0.69–11.61) (*n* = 23)	0.81 (0.05–153.50)	0.322/0.564/r = 0.21

The *p* and q values represent within-group preoperative–postoperative comparisons; q values are FDR-adjusted separately within each group. Exact paired n is shown in the preoperative cell. Values are mean ± SD or median (min–max), as appropriate.

## Data Availability

The data supporting the findings of this study are available from the corresponding author upon reasonable request, subject to institutional and ethical restrictions.

## Abbreviations

The following abbreviations are used in this manuscript: CBC, complete blood count; CRP, C-reactive protein; FDR, false discovery rate; FNA, fine-needle aspiration; fT3, free triiodothyronine; fT4, free thyroxine; Hb/RDW, hemoglobin-to-red blood cell distribution width ratio; LMR, lymphocyte-to-monocyte ratio; MCV, mean corpuscular volume; NLR, neutrophil-to-lymphocyte ratio; PLR, platelet-to-lymphocyte ratio; PTC, papillary thyroid carcinoma; RDW, red blood cell distribution width; TSH, thyroid-stimulating hormone.
